# Horner's Syndrome as Initial Manifestation of Possible Brachial Plexopathy Neurolymphomatosis

**DOI:** 10.3389/fneur.2019.00004

**Published:** 2019-01-22

**Authors:** Wijdan Rai, Vanessa Olcese, Bakri Elsheikh, Amro Maher Stino

**Affiliations:** ^1^Department of Neurology, Ohio State University Wexner Medical Center, Columbus, OH, United States; ^2^Department of Neurosurgery, Ohio State University Wexner Medical Center, Columbus, OH, United States

**Keywords:** lymphoma, Horner's syndrome, brachial plexopathy, non-Hodgkin's lymphoma, diffuse large B-cell lymphoma, CSF

## Abstract

**Introduction:** Horner's syndrome is an established clinical finding unique to neoplastic brachial plexopathy.

**Background:** We present the case of a patient who developed Horner's syndrome as the first manifestation of neurolymphomatosis (NL) of the brachial plexus that did not have the usually associated bulky adenopathy/Pancoast syndrome phenotype.

**Discussion:** We discuss the clinical utility of Horner's syndrome with regards to brachial plexopathy of indeterminate etiology, as well as the utility of other diagnostic modalities in NL.

**Concluding Remarks:** NL, particularly of the brachial plexus, is particularly challenging to diagnose. MRI and CSF studies are often inconclusive. FDG-PET imaging can be difficult to get insurance to approve. The presence of Horner's syndrome in brachial plexopathy of indeterminate etiology, even in the absence of bulky adenopathy, should raise clinical suspicion of NL, possibly prompting such interventions as fascicular nerve biopsy.

## Introduction

Neurolymphomatosis (NL) is characterized by direct lymphomatous cell infiltration of peripheral nerves, nerve roots, or plexi in the context of non-Hodgkin's lymphoma (1) NL is a very common manifestation of diffuse large B cell lymphoma. Although NL has been well characterized, establishing its diagnosis in the context of brachial plexopathy is difficult. MRI findings alone are non-specific (2) and although the diagnosis can be supported by CSF testing, CSF usually lacks sensitivity (3). The diagnosis is confirmed by histological demonstration of NL in a tissue sample or through concordant PET and MR imaging. Thus, any findings that alert the clinician to the possibility of a neoplastic etiology are invaluable. The utility of Horner's syndrome in distinguishing neoplastic from radiation-induced plexopathy has been well-described, (4) albeit in the setting of bulky adenopathy (namely Pancoast syndrome phenotype). Here, we present the case of a young woman with presumptive NL involving the brachial plexus. Ipsilateral Horner's syndrome was one of her first presenting features; however, there was no evidence of extrinsic compressive bulky adenopathy at any point in the disease course, thus representing a unique subset of NL plexopathy separate from Pancoast syndrome.

## Background

The patient was a 25 year old female nursing student with personal history of asthma. She reported no smoking history; she had a family history of lupus in a paternal aunt, celiac in a brother, and Crohn's in a sister. She provided written informed consent for participation and publication of this case report. Starting abruptly in early October 2015, she noticed left eye droop and lack of forehead sweating after working out. She also reported tingling in her fourth and fifth left digits. She presented to an outside institution and was diagnosed with idiopathic inflammatory plexopathy (Parsonage Turner), for which a prednisone taper was started. Electrodiagnostic (EDX) testing had shown a lower trunk brachial plexopathy. MRI of the brachial plexus with and without contrast was negative. Serologic testing showed a positive ANA 1:160 (speckled), with a negative ENA screen. Late in December 2015, she developed acute onset left axillary pain with arm weakness along with worsening headache and was admitted to our institution. Neurologic examination at the time showed left ptosis with associated miosis (1.5 mm smaller on the left than on right) with left arm strength as follows (per Medical Research Council grading): deltoid grade IV; biceps II; triceps II; wrist extensors; wrist flexors; and finger flexors IV. Sensory testing revealed decreased sensation to pinprick and light touch in the medial forearm. CSF evaluation showed an elevated white blood cell (WBC) count (18) with reported lymphocytosis (81%) with typical cells, slightly elevated protein levels (48 mg/dl; normal range 15–45 mg/dl), and normal glucose (68 mg/dl). No oligoclonal bands were seen and the IgG index was normal (0.53; normal <0.66). CSF cytology, herpes zoster polymerase chain reaction (PCR), and paraneoplastic panel were all negative. At this time, repeat EDX showed pan-plexopathy of the left brachial plexus, predominantly in the lower trunk (Table 1). Specifically, there was evidence of an asymmetrically lower sensory nerve action potential (SNAP) amplitude in the left ulnar nerve, as well as an absent SNAP in the left medial antebrachial cutaneous nerve. The median and ulnar compound muscle action potential (CMAP) responses were low amplitude. Needle EMG evaluation showed fibrillation potentials in the first dorsal interosseous, extensor digitorum, and flexor digitorum profundus 4,5 muscles, with neurogenic units seen in all 3 muscles as well as the biceps, brachial radialis, and flexor carpi ulnaris muscles. MRI of the brain and cervical spine showed no enhancement. CT chest imaging was negative for adenopathy and/or tumors. Though clinical suspicion of lymphoma was high, the lack of objective data (as per insurer's requirements) to support a diagnosis made it impossible to get approval for a FDG-PET. Fascicular nerve biopsy of the brachial plexus (not offered at our institution) was also considered, but given the lack of enhancement or hyperintensity on MR plexus imaging, it was unclear what segment of the plexus to target. In light of the patient's significant refractory pain, the absence of conclusive objective findings to support a diagnosis of lymphoma, and reasonable concern for an autoimmune or inflammatory etiology (e.g., macrovasculitic plexopathy), it was decided to administer 1 g of intravenous methylprednisolone daily for 5 days. She was also started on amitriptyline, gabapentin, oxycodone, and ibuprofen. The patient reported no B symptoms, specifically no complaints of fever, weight loss, or night sweats at any point in time in her clinical course. At the time of discharge, there was improvement in the triceps and biceps (grade IV and III, respectively), with associated improvement in pain.

Table 1Nerve conduction studies and EMG findings demonstrating left lower trunk predominant brachial plexopathy.

**Table:** 

**Nerve/Sites**	**Rec.** ** Site**	**Onset** ** ms**	**Peak** ** ms**	**SNAP Amp** ** μV**	**Dist** ** cm**	**Vel** ** m/s**
**L Median—Dig II**						
Dig II	Wrist	2.08	2.66	34.6	12	57.6
Palm	Wrist	1.41	1.88	78.6	8	56.9
**R Median—Dig II**						
Dig II	Wrist	1.98	2.55	34.7	11	55.6
Palm	Wrist	1.51	1.98	69.9	8	53.0
**L Ulnar—Dig V**						
Dig V	Wrist	1.93	2.50	**10.8**	10	51.9
Palm	Wrist	1.56	2.14	11.8	8	51.2
**R Ulnar—Dig V**						
Dig V	Wrist	1.77	2.29	20.6	10	56.5
Palm	Wrist	1.46	1.93	40.1	8	54.9
**L Radial**						
Forearm	Snuffbox	1.88	2.50	**12.0**	11	58.7
**R Radial**						
Forearm	Snuffbox	1.72	2.29	43.5	11	64.0
**L AnteBr Cut**						
Lateral	Forearm	2.24	2.76	11.9	12	53.6
Medial	Forearm	**NR**	**NR**	**NR**		
**R AnteBr Cut**						
Lateral	Forearm	2.92	3.49	14.5	10	34.3
Medial	Forearm	2.14	3.02	20.5	11	51.5

**Table:** 

**Nerve/Sites**	**Rec. Site**	**Lat.** ** ms**	**Amp** ** mV**	**Dist** ** cm**	**Vel** ** m/s**
**L Median—APB**					
Wrist	APB	3.39	**2.5**	7	
Elbow	APB	7.34	**2.2**	20	50.5
**L Ulnar—ADM**					
Wrist	ADM	3.02	**2.0**	7	
B. Elbow	ADM	6.09	**1.9**	17	55.3
A. Elbow	ADM	7.92	**1.8**	10	54.9

**Table:** 

**EMG summary table**													
	**SA**				**Amp**		**Dur**		**PolyP**	**Rate**		**Recr**	**Comment**
	**IA**	**Fib**	**PSW**	**Fasc**	**#**	**–**	**#**	**–**	**–**	**#**	**–**	**Pattern**	**–**
L Deltoid	Nl	0	0	None	All	Nl	Many	LN	Many	Few	Fast	Dec	
L Biceps	Nl	0	0	None	All	Nl	Few	LN	Few	Few	Fast	Dec	
L Triceps	Nl	0	0	None	All	Nl	All	Nl	Nl	All	Nl	Nl	Nl
L Pronator Teres	Nl	0	0	None	All	Nl	All	Nl	Nl	All	Nl	Nl	Nl
L Brachial Radialis	Nl	0	0	None	All	Nl	Few	LN	Many	Few	Fast	Dec	
L Flexor Digitorum Profundus 2,3	Nl	0	0	None	All	Nl	Few	LN	Many	Few	Fast	Dec	
L Flexor Digitorum Profundus 4,5	Nl	1+	1+	None	All	Nl	All	Nl	Many	Many	Fast	Dec	
L Flexor Carpi Ulnaris	Nl	2+	2+	None	All	Nl	Many	LN	Many	Few	Fast	Dec	
L Extensor Digitorum Communis	Nl	1+	1+	None	All	Nl	Many	LN	Many	Few	Fast	Dec	
L Flexor Digitorum Indicis	Nl	2+	2+	None	All	Nl	Many	LN	Nl	Few	Fast	Dec	

*Dig II, digit II; Dig V, digit V; AnteBr Cut, antebrachial cutaneous; Rec, recording site; Onset, onset latency; Peak, peak latency; SNAP, sensory nerve action potential; Amp, amplitude; Dist, distance; Vel, velocity; NR, no response; APB, abductor pollicis brevis; ADM, abductor digiti minimi; Lat, latency; SA, spontaneous activity; Dur, duration; PolyP, polyphasic; Recr, recruitment; IA, insertional activity; Fib, fibrillation potentials; PSW, positive sharp waves; Fasc, Fasciculation potentials; Nl, normal; LN, long; Dec, decreased*.

Weakness recurred 2 weeks later, however, with the patient stating that she could no longer flex her biceps; she also reported increased shoulder pain. She was re-evaluated in clinic and underwent repeat CSF evaluation, which showed an elevated WBC count of 9 (reactive lymphocytosis noted) and normal protein levels (39 mg/dl). Due to progressive weakness and refractory pain, she was electively readmitted in late January 2016. Examination revealed persistent ptosis and miosis on the left side, with left arm strength as follows: deltoid grade II; triceps IV; biceps I; wrist extensors, wrist flexors, and interossei IV; with absent left biceps and brachoradialis reflexes. Sensory testing showed hyperesthesia to light touch in the proximal lateral arm and diminished sensation to pinprick was noted distally. Repeat high volume CSF testing showed elevated WBC count (11) with normal protein levels (43). Lymphocytosis (83%) was noted in the presence of large atypical lymphocytes. MRI of the left brachial plexus was also repeated, which showed diffuse hyperintensity with no enhancement. Hematology was consulted to evaluate for CNS lymphoma; CSF B and T cell rearrangement tests and cytology were normal. The patient received another 5 days of intravenous methylprednisolone for a putative diagnosis of progressive inflammatory brachial plexopathy, with modest interval improvement in strength, with the biceps, deltoid, and infraspinatus all improved from no antigravity to at least a grade III. Outpatient evaluation in February 2016 revealed persistent left ptosis and miosis (left pupil 3 mm, right pupil 4.5 mm), with left arm strength as follows: deltoid grade III; triceps V; biceps III; wrist extensor V; and finger flexors, finger extensors, APB, and interossei IV. Of note, the triceps reflex was absent. She continued to report refractory pain and subjective decline in strength.

**Figure 1:**
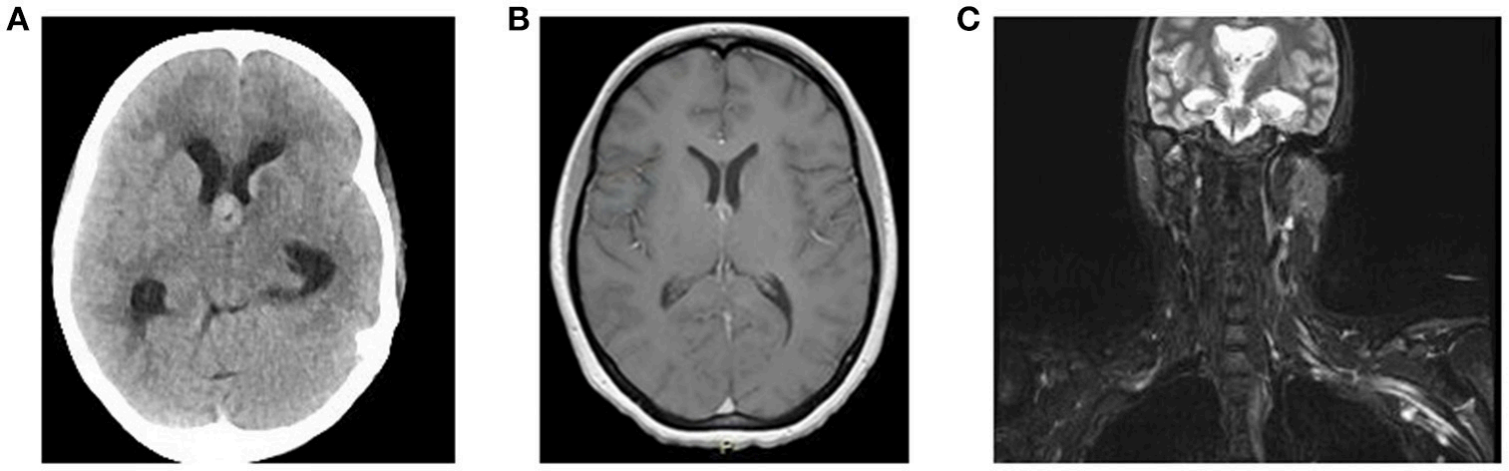
**(A)** Non-contrast CT Head demonstrates a lesion at the Foramen of Monro causing obstructive hydrocephalus. **(B)** MRI brain T1 axial study with contrast demonstrates enhancement of lesion at the Foramen of Monro. **(C)** MRI brachial plexus STIR sequence shows diffuse enlargement with marked T2 hyperintensity of the left brachial plexus.

**Figure 2:**
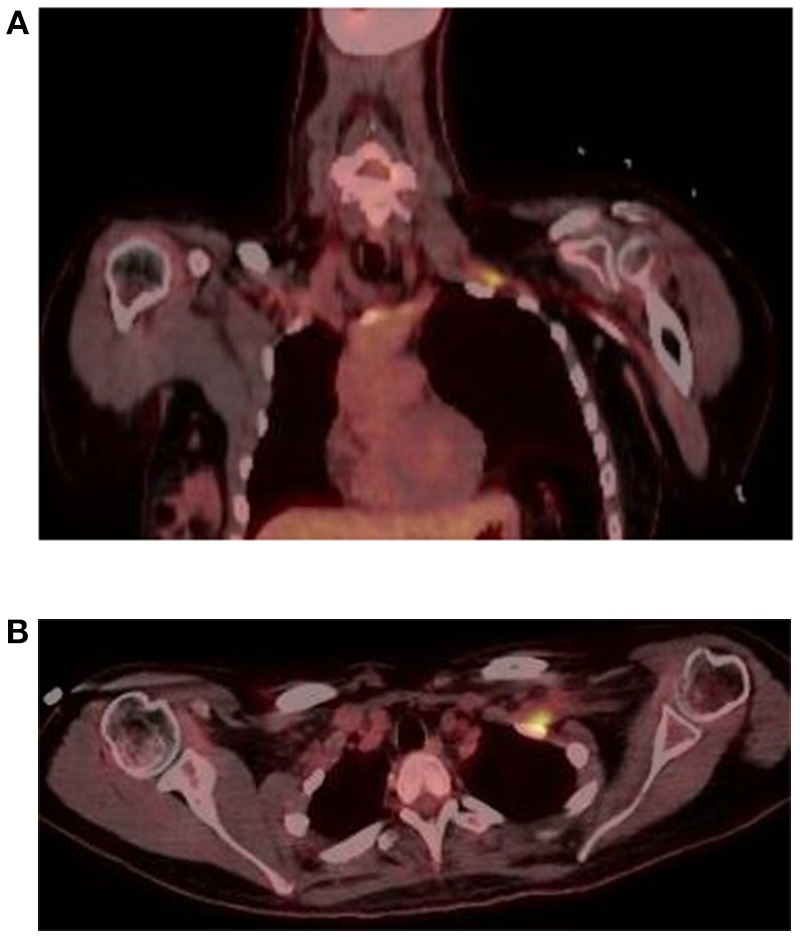
FDG PET coronal **(A)** and axial **(B)** sequences show focal hyperactivity at the left pectoral/subpectoral level adjacent to the first rib.

In June 2016, she was brought to the emergency room by her family, who reported increased confusion, nausea, vomiting, headache, and urinary incontinence. She was disoriented and unable to follow commands. Bilateral knee and ankle hyperreflexia (grade 3) was also noted. CT of the head revealed a third ventricular hemorrhage causing obstructive hydrocephalus (Figure 1A). The neurosurgery team placed an emergent right frontal external ventricular drain and performed a 4 vessel angiogram, which excluded aneurysm or arterial malformation. MRI of the brain revealed multiple enhancing lesions in the subependymoma with diffusion restriction and nodularity in the septum pellucidum, caudate, hypothalamus, and foramen of Monro, with findings concerning for a leptomeningeal neoplastic process (Figure 1B). MRI of the cervical, thoracic, and lumbar spine regions showed contrast enhancement of the C4 through C8 nerve roots. Repeat MRI of the brachial plexus showed enhancement from nerve roots into distal branches in addition to diffuse hyperintensity seen on prior imaging (Figure 1C). Repeat CT of the chest and abdomen/pelvis was normal, with no evidence of lymphadenopathy. Given the above findings suggestive of lymphoma, whole body FDG-PET imaging was approved and completed, with evidence of focal activity seen in the left pectoral/subpectoral region adjacent to the first rib in addition to hypermetabolic brain lesions (Figures 2A,B). Repeat CSF evaluation showed <3 WBC count, with protein levels ranging from 56 to 70 mg/dl. Large atypical lymphocytes were again noted. CSF immunophenotyping (previously negative) now showed a lambda restricted B cell population (CD5-, CD10-, C-myc negative, IGH/BCL-2 negative, bcl-6 positive) and the patient was diagnosed with stage IV diffuse large B cell lymphoma. Bone marrow immunophenotyping was negative; ophthalmological evaluation showed no ocular involvement. In the context of definite lymphoma, the patient was finally started on chemotherapy. She ultimately received 6 cycles of pulsed intravenous dexamethasone and R-CHOP with methotrexate. Upon re-assessment in October 2016, repeat MRI of the brain showed complete resolution of enhancement and lesions. MRI of the brachial plexus also showed significant improvement in T2 hyperintensity and enhancement. Repeat neurologic exam showed resolution of her left ptosis and miosis. Formal muscle testing of the left upper limb showed normal strength with the return of her left biceps, brachoradialis, and triceps reflexes. The patient also reported complete resolution of pain. The following month, she completed an autologous stem cell transplant with Thiotepa and BNCU conditioning. Restaging scans showed complete remission, which has been maintained as of her last outpatient visit in August 2018.

## Discussion

The diagnosis of NL in the context of brachial plexopathy can be challenging, particularly given the lack of sensitivity and/or specificity of standard imaging and CSF studies. Furthermore, most medical centers do not have the capacity to perform fascicular nerve biopsy of the brachial plexus (gold standard). Although no histological confirmation of NL was obtained in our patient through fascicular nerve biopsy of the brachial plexus, multiple factors were highly suggestive of the diagnosis. She had focal hyperactivity on PET imaging, hyperintensity and enhancement on MR imaging, and a lambda-restricted B cell population on CSF evaluation. Our patient's final diagnosis was stage IV diffuse large B cell lymphoma with CNS involvement, as the brachial plexus was initially involved.

In this case, we highlight the utility of Horner's syndrome as a first finding of possible NL brachial plexopathy, even in the absence of bulky adenopathy / Pancoast Syndrome (Table 2). To reiterate, CT chest imaging studies of our patient showed no bulky adenopathy. Horner's syndrome has been well-described in NL, but almost always in the setting of extrinsic compressive lesions (4, 5) NL more commonly involves proximal segments of the brachial plexus but does not have a predilection for the lower trunk, as was seen in our patient (6). Refractory pain and positive sensory symptoms (such as hyperesthesias in our patient) are also suggestive of lymphomatous plexopathy (1, 4). Stepwise progression of disease burden, as evidenced by worsening muscle strength, should also raise concern for lymphomatous plexopathy (7). While lymphoma was suspected all along in our case, the presence of Horner's syndrome further supported the presence of an infiltrative neoplastic process, such as NL.

**Table 2:** Radiologic, CSF, and exam findings over time in patient.

	**Oct 2015**	**Dec 2015**	**Early Jan 2016**	**Late Jan 2016**	**June 2016**	**Oct 2016** ** (post-R-CHOP)**
PET		Denied by insurance			Focal activity L pectoral/ subpectoral region + brain	
CT chest		Negative			Negative	
**MRI**						
Brain		Negative			Multiple enhancing lesions	Complete resolution
Spine		Negative			Enhancing left C4-C8 Roots	
**MRI BRACHIAL PLEXUS**						
T2 hyperintensity	Negative			Diffusely hyperintense	Diffusely hyperintense	Reduction
T1 enhancement	Negative			Negative	Yes, from roots into distal branches	Complete resolution
**CSF**						
WBC		18 (81% lymphocytosis)	9	11 (83% lymphocytosis)	<3	
Protein (mg/dL)		48	39	43	56, 70	
Cytology		Negative	Reactive lymphocytosis	Lymphocytosis with atypical large lymphocytes	Large atypical lymphocytes	
Immunophenotype		Negative		B and T Cell Rearrangement studies negative	Lambda restricted B cell population	
**EXAM/SYMPTOMS**						
Horner's syndrome	Present	Present	Present	Present		Resolved
Left Arm strength		Deltoid IV, biceps/triceps II, WE/WF/FF IV		Deltoid II, Triceps IV, Biceps now I, WE/WF/IO IV		Normal
Steroid responsiveness		Partial improvement in strength		Partial improvement in strength		
Pain	Tingling digits IV and V	Axillary pain	Shoulder pain			Resolved
EMG/NCS	Left lower trunk plexopathy	Left pan- plexopathy, lower trunk predominant				

*PET, positron emission tomography; MRI, magnetic resonance imaging; CSF, cerebrospinal fluid; WBC, white blood count; EMG/NCS, electromyography nerve conduction studies, WE, wrist extensors; WF, wrist flexors; FF, finger flexors; IO, interossei*.

The pathophysiology of NL consists of direct peripheral nerve infiltration, hematogenous spread, and intravascular proliferation. Lymphocytes are also known to infiltrate nerves adjacent to lymph nodes, commonly at the level of the dorsal root ganglia due to deficiency of the blood-nerve barrier at that level. Lymphomatous cells have been shown to directly infiltrate all of the epineurium, endoneurium, (8) and perineurium (9). In our patient, who did not have lymphadenopathy, Horner's syndrome was likely caused by lymphomatous invasion of the superior cervical ganglion through the connecting gray rami at the C8-T1 level. We suspect infiltration of the pre-ganglionic second order neuron between the spinal center of Budge-Waller and the superior cervical ganglion. At the time of diagnosis, she did not show CNS involvement on MR imaging, nor were there upper motor neuron findings to suggest first order involvement. Furthermore, there was no evidence of cervical lymphadenopathy to suggest third order involvement. However, she did not have formal ophthalmologic pharmacologic testing to distinguish second from third order neuron involvement.

While there have been reports of Horner's syndrome associated with diffuse large B cell lymphoma, (10) non-Hodgkin's lymphoma, (11) and extrapulmonary lymphoid granulomatosis, (12) most have been in the context of bulky adenopathy. The literature on Horner's syndrome in NL plexopathy is no different (13–15). Our case represents a rarer subtype of patients who have Horner's syndrome without bulky adenopathy. A 2017 report by Abascal et al. documented the onset of ptosis and miosis in a 19 year old male as the initial manifestation of Hodgkin's lymphoma, although the impact of mediastinal adenopathy on the sympathetic chain was unclear (article in Spanish) (16). A separate report described Horner's syndrome in a 35 year old female with cervical adenopathy causing a third order lesion, as confirmed on phenylephrine testing (17). Peltier reported a case of Horner's syndrome from intramedullary spinal cord lymphoma, representing first order injury (18).

MRI and FDG-PET are reported to have 80 and 85–90% specificity, respectively, in identifying lymphomatous plexopathy. Contrast enhancement and focal nerve thickening do not necessarily suggest malignancy (2). Although FDG PET is more sensitive, it is often denied by insurance if the patient does not already have an established diagnosis of lymphoma, as in our case. Furthermore, negative cases of whole body PET have been reported in patients who are later found to have biopsy-proven NL plexopathy (7).

The sensitivity of CSF studies at detecting lymphoma is reported as no higher than 80%, even if up to 3 taps are done (3). CSF protein elevation is only 61% sensitive for CNS lymphoma (1). In addition, normal WBC count does not preclude the presence of malignant cells (1). Our patient had normal WBC (<3) on multiple taps. Even when elevated WBC count was noted, cytologic evaluation was inconclusive. Initial immunophenotyping and gene rearrangement studies were also negative, further adding to our diagnostic challenge.

## Concluding Remarks

Although preliminary testing may be negative or inconclusive, the presence of unexplained Horner's syndrome in the setting of unexplained brachial plexopathy should prompt consideration for fascicular nerve biopsy testing to evaluate for NL. Unique to our case is that such a Horner's syndrome occurred in the absence of bulky adenopathy, as is typical with Pancoast Syndrome.

## Author Contributions

AS and BE contributed to manuscript rationale and development. AS, VO, and WR were involved in patient management, identification, and manuscript development.

### Conflict of Interest Statement

The authors declare that the research was conducted in the absence of any commercial, or financial relationships that could be construed as a potential conflict of interest.

## References

[1] Grisariu S Avni B Batchelor TT Van Den Bent MJ Bokstein F Schiff D. Neurolymphomatosis: an international primary CNS lymphoma collaborative group report. Blood (2010) 115:5005–11. 10.1182/blood-2009-12-258210 20368468 PMC3710441

[2] Swarnkar A Fukui MB Fink DJ Rao GR. MR imaging of brachial plexopathy in neurolymphomatosis. AJR Am J Roentgenol. (1997) 169:1189–90. 10.2214/ajr.169.4.9308489 9308489

[3] Wasserstrom WR Glass JP Posner JB. Diagnosis and treatment of leptomeningeal metastases from solid tumors: experience with 90 patients. Cancer (1982) 49:759–72. 6895713 10.1002/1097-0142(19820215)49:4<759::aid-cncr2820490427>3.0.co;2-7

[4] Lederman RJ Wilbourn AJ Brachial plexopathy recurrent cancer or radiation? Neurology (1984) 34:1331–5. 10.1212/WNL.34.10.1331 6090988

[5] Kori SH Foley KM Posner JB. Brachial plexus lesions in patients with cancer: 100 cases. Neurology (1981) 31:45–50. 10.1212/WNL.31.1.45 6256684

[6] Harper CM Thomas JE Cascino TL Litchy WJ Distinction between neo plastic and radiation-induced brachial plexopathy, with emphasis on the role of EMG. Neurology (1989) 39:502–6. 10.1212/WNL.39.4.502 2538777

[7] Lahoria R Dyck PJ Macon WR Crum BA Spinner RJ Amrami KK. Neurolymphomatosis: a report of 2 cases representing opposite ends of the clinical spectrum. Muscle Nerve (2015) 52:449–54. 10.1002/mus.24646 25758704

[8] Purohit DP Dick DJ Perry RH Lyons PR Schofield IS Foster JB. Solitary extranodal lymphoma of sciatic nerve. J Neurol Sci. (1986) 74:23–34. 10.1016/0022-510X(86)90188-7 3723134

[9] Tomita M Koike H Kawagashira Y Iijima M Adachi H Taguchi J. Clinicopathological features of neuropathy associated with lymphoma. Brain (2013) 136:2563–78. 10.1093/brain/awt193 23884813

[10] Lueangarun S Auewarakul CU. Diffuse large B cell lymphoma presenting as Horner's syndrome in a patient diagnosed with neurofibromatosis type 1: a case report and review of the literature. J Med Case Rep. (2012) 6:8. 10.1186/1752-1947-6-8 22236362 PMC3314538

[11] Mills PR Han LY Dick R Clarke SW. Pancoast syndrome caused by a high grade B cell lymphoma. Thorax (1994) 49:92. 10.1136/thx.49.1.92 8153951 PMC474122

[12] Dolan G Smith J Reilly JT. Extrapulmonary lymphomatoid granulomatosis presenting as Pancoast's syndrome. Postgrad Med J. (1991) 67:914–5. 10.1136/pgmj.67.792.914 1661891 PMC2399176

[13] Rao RD Robins HI. Non-Hodgkin's tumor and pancoast's syndrome. Oncol Rep. (2001) 8:165–71. 10.3892/or.8.1.165 11115591

[14] Alla Dolganova AL Barra M da Silva Moreira J Síndrome de Pancoast causada por linfoma. J Pneumol. (2000) 26:145 10.1590/S0102-35862000000300009

[15] Simon SR Dorighi JA Branda RF Ershler WB Homer's syndrome: an unusual presentation of Hodgkin's disease. Med Pediatr Oncol. (1985) 13:390–1. 10.1002/mpo.2950130618 4046977

[16] Abascal CA Abarzuza RC Plaza PR Horner's Syndrome: an unusual ophthalmological presentation of Hodgkin lymphoma. An Sist Sanit Navar. (2017) 40:461–6. 10.23938/ASSN.0117 29215661

[17] Ruiz E Resende LS, Gaiolla RD, Niéro-Melo L, Domingues MA, de Lima Resende LA. Post-ganglionic Horner's syndrome: an unusual presentation of non-Hodgkin lymphoma. Case Rep Neurol. (2012) 4:43–6. 10.1159/000335521 22611367 PMC3355647

[18] Peltier J Cretu I Fichten A Toussaint P Desenclos C Le DG. Primary intramedullary lymphoma. Case report. Neurochirurgie (2007) 53:375–8. 10.1016/j.neuchi.2007.06.002 17689569

